# How do state Medicaid programs determine what substance use disorder treatment medications need prior authorization? An overview for clinicians

**DOI:** 10.1186/s13722-020-00194-7

**Published:** 2020-06-29

**Authors:** Marcus A. Bachhuber

**Affiliations:** Section of Community and Population Medicine, Department of Medicine, Louisiana State University Health Sciences Center-New Orleans, 533 Bolivar Street, 5th Floor, New Orleans, LA 70112 USA

**Keywords:** Medicaid, Prescription drugs, Drug costs, Opioid-related disorders, Prior authorization, Healthcare financing

## Abstract

The process by which state Medicaid programs develop their preferred drug lists, and determine which medications require prior authorization, is opaque to many clinicians. This process is a synthesis of cost and clinical information. For cost, the federal Medicaid Drug Rebate Program establishes mandatory rebates that pharmaceutical manufacturers must pay state Medicaid programs. In addition, state Medicaid programs may also negotiate supplemental rebates whereby, in exchange for a preferred position on the preferred drug list, manufacturers pay an additional rebate. These supplemental rebates are most important in therapeutic classes with multiple brand competitors (e.g., medication treatments for opioid use disorder). For clinical information, state Medicaid programs convene pharmaceutical and therapeutics committees, drug utilization review boards, or both, composed of a variety of stakeholders such as practicing clinicians. Cost factors such as federal rebate calculations and supplemental rebate negotiations may lead to counterintuitive preferred drug lists, for example, a state Medicaid program requiring prior authorization for a generic medication but not for its brand equivalent (e.g., buprenorphine/naloxone products). Because of states’ reliance on rebates, mandates to remove prior authorization may have the unintended consequence of increasing costs significantly through the loss of rebate negotiating power. In the face of high and rising medication costs, state Medicaid programs are also implementing innovative policy approaches to maintain access and control costs, such as targeted rebate negotiation and value-based pricing. Through participation in state Medicaid program clinical advisory committees, individual clinicians can have a powerful voice. Interested clinicians should consider joining to inform policy and help ensure their patients’ needs are met.

Case: A 34-year-old man who is currently injecting heroin daily presents as a new patient to your clinic. On interview, the patient meets criteria for severe opioid use disorder and expresses an interest in starting treatment. As a primary care provider trained in office-based opioid use disorder treatment, you recommend buprenorphine/naloxone as a first-line treatment option. After a detailed discussion of the risks and benefits, the patient agrees, and elects an at-home induction. You send a prescription for generic buprenorphine/naloxone film to the pharmacy next door.

Fifteen minutes later, the pharmacist calls and tells you that generic buprenorphine/naloxone film requires a prior authorization under the patient’s Medicaid plan. Interestingly, the brand film is covered without prior authorization. You know that generic medications are equally effective and less expensive than brand medications and so you are left wondering: Why would a state Medicaid program, with limited resources, prefer a brand medication over a generic medication?

## The Medicaid Drug Rebate Program

While high and rising prescription medication costs have received considerable media attention in recent years, the topic has been a longstanding concern of federal and state policymakers. Established by the Omnibus Budget Reconciliation Act of 1990, the federal Medicaid Drug Rebate Program (MDRP) has, for decades, been the primary mechanism to contain costs. Because of its role in cost containment, the MDRP is the framework underlying administration of Medicaid pharmacy benefits nationally. In short, after Medicaid programs reimburse pharmacies for dispensed medications, the MDRP provides for a mandatory rebate, commonly called the federal rebate, which pharmaceutical manufacturers must then pay to states. The MDRP controls both state and federal costs because rebates received by states are subsequently shared with the federal government according to states’ Federal Medical Assistance Percentage [1].

While prices and rebate amounts are confidential under federal law, the methodology used to calculate the federal rebate is public [2]. For brand medications, the federal rebate is calculated as a base rebate which is the greater amount of: (1) 23.1% of the average manufacturer price, or (2) average manufacturer price minus “best price.” (Best price refers to the best price a manufacturer has given to most other payers, with some limited exceptions for certain federal health care programs [3]). In addition to the base rebate, there is an additional inflationary rebate added for an increase in the average manufacturer price faster than the Consumer Price Index Urban value, if applicable. For generic medications, the federal rebate is calculated as a base rebate of 13% of the average manufacturer price plus the inflationary rebate, if applicable. There are many additional nuances to the program, in particular how medications are classified as brand or generic, described in detail elsewhere [4, 5].

The MDRP is successful in obtaining rebates for state Medicaid programs that are substantially higher than what other payers receive: 51% of gross expenditures in Medicaid relative to 22% for Medicare Part D plans and 12% for commercial plans [6]. In addition, the MDRP also ensures that Medicaid members have access to virtually all outpatient prescription drugs because, if a manufacturer signs a rebate agreement, the medication must be payable (i.e., true closed formularies are not currently allowed). Further, the MDRP underlies cost containment for other large federal programs because signing a rebate agreement for Medicaid also requires a manufacturer to provide discounted medications to Department of Veterans Affairs programs and the 340B Drug Pricing Program.

## Rebates and the Medicaid preferred drug list

In addition to federal rebates under MDRP rules, state Medicaid programs also frequently negotiate supplemental rebates for brand medications. While removing a medication from the formulary and making it non-payable is not allowed, Medicaid programs do have the flexibility to create and maintain preferred drug lists. In exchange for a supplemental rebate, Medicaid programs can offer manufacturers a preferred position on their preferred drug list, meaning that the medication can generally be dispensed without prior authorization and is therefore likely to be favored by prescribers. This preferred position can take the form of an exclusive position as the sole preferred medication in a therapeutic class, or as one of two or more preferred medications. State Medicaid programs can negotiate their own supplemental rebates or join multi-state pools to increase bargaining power [7].

**Fig. 1:**
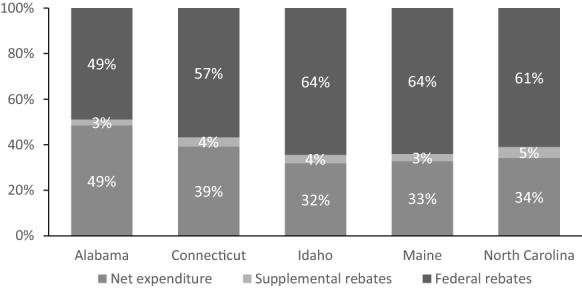
Medicaid prescription medication rebates, by type, and resulting net expenditure, 2017. Refers to net expenditures of state and federal matching funds

Federal and supplemental rebates are critical state and federal cost offsets to Medicaid prescription medication expenditures (Table 1). Figure 1 illustrates expenditure data from 5 example states, taken from federal reporting [8]. Each column represents states’ gross prescription medication expenditures, normalized to 100%, with the corresponding percentage received back from manufacturers, by type of rebate, and the resulting percentage net expenditure. Although head-to-head comparisons of states are not possible due to differences in member needs and comorbidities as well as regional prescribing patterns, federal rebates offset more than half of gross expenditures. While the overall offset of supplemental rebates is relatively small as a proportion of gross expenditures, they are important in controlling costs for classes with multiple brand alternatives (e.g., stimulants for attention deficit/hyperactivity disorder). Further, given the overall magnitude of gross pharmacy expenditures, state supplemental rebates are substantial in absolute terms (e.g., approximately $88.8 million in North Carolina).

**Table 1:** Types of rebates available to state Medicaid programs under the Medicaid Drug Rebate Program

Rebate type	Entity collecting the rebate	Brand versus generic	Methodology
Federal rebate, also known as mandatory or statutory rebate	State Medicaid program	Brand	Base rebate, the greater of: 23.1% of the average manufacturer price, or Average manufacturer price minus “best price”^a^ Plus the inflationary rebate, if applicable: Difference between the increase in average manufacturer price and the increase in Consumer Price Index Urban value
		Generic	Base rebate: 13% of the average manufacturer price Plus the inflationary rebate, if applicable: Difference between the increase in average manufacturer price and the increase in Consumer Price Index Urban value
Supplemental rebate	State Medicaid program or Medicaid managed care plans, varies by state	Brand only	Methodology is proprietary and confidential Rebates are typically provided in exchange for a preferred position on the preferred drug list

^a^“Best price” refers to the best price a manufacturer has given to most other payers, with some limited exceptions for certain federal health care programs

## Clinical considerations for the preferred drug list

Without question, the preferred drug list drives prescribing to Medicaid members. Therefore, while federal and supplemental rebates guide its creation, clinical concerns are fundamental. State Medicaid programs typically operate pharmaceutical and therapeutics committees consisting of various stakeholders that weigh rebate information with clinical knowledge of efficacy and safety. For example, clinical committee members can ensure that preferred medications have an efficacy and safety profile that is at least as favorable as alternative medications. In addition, clinical committee members can bring knowledge of local practice patterns. For example, in some cases, the net cost after rebates for a certain medication in a therapeutic class may be far lower than its competitors. However, if prescribers are not likely prescribe it, there are several problems. Prescribers will now have to get prior authorization for the more commonly used medication, patients may have their therapy delayed, and the state may be better off financially by preferring the more commonly prescribed medication and negotiating as high a supplemental rebate as possible.

In some states with Medicaid managed care programs, managed care organizations’ pharmacy benefits managers negotiate and collect their own supplemental rebates and operate their own pharmaceutical and therapeutics committees. This can result in multiple disparate preferred drug lists across the Medicaid program, creating administrative burden for prescribers. To alleviate this burden, several states have moved toward a uniform preferred drug list for all managed care organizations and the state fee-for-service program, if applicable. For example, until recently in Louisiana Medicaid, there were 6 distinct preferred drug lists: 1 for each of the 5 managed care organizations and 1 for the fee-for-service program. While the preferred drug lists overlapped, there were significant differences in several key classes with multiple competitor brand medications (e.g., insulins). In May 2019, Louisiana Medicaid transitioned to a single, uniform, preferred drug list across the entire program. The state now negotiates and collects all supplemental rebates for medications dispensed to all Medicaid members and the state’s pharmaceutical and therapeutics committee guides creation of the preferred drug list for the entire program.

In addition to preferred and non-preferred status, Medicaid programs sometimes require a prior authorization for an entire therapeutic class, often termed a clinical prior authorization. This is typically reserved for classes that are high risk (e.g., psychiatric medications for children) or high cost (e.g., direct acting antivirals for hepatitis C). Then, within the class, there remain preferred and non-preferred medications. Clinical prior authorization requirements, along with other clinical edits on pharmacy claims (e.g., age restrictions, quantity limits, and checks for therapeutic duplication) are typically reviewed and recommended by federally-mandated state drug utilization review boards, which are advisory boards composed of prescribers and other stakeholders. In some states, pharmaceutical and therapeutics committee functions and drug utilization review board functions are combined into one committee.

## Pricing for brand versus generic medications

Because of MDRP rules and supplemental rebates, the net cost to the state for generic medications may be counterintuitive, especially for generic medications that were recently only available as a brand. A common of example of this is buprenorphine/naloxone. While Medicaid’s upfront reimbursement to pharmacies for a dispensed generic medication will be lower than the reimbursement for the corresponding brand, the state will generally receive a lower federal rebate on the generic medication due to a lower base rebate percentage and lack of best price protection. Furthermore, any supplemental rebate negotiated for the brand medication does not apply to the generic medication and will be lost. In total, loss of rebates can lead to a substantially higher net expenditure, after rebates, for a generic medication relative to its brand equivalent.

**Fig. 2:**
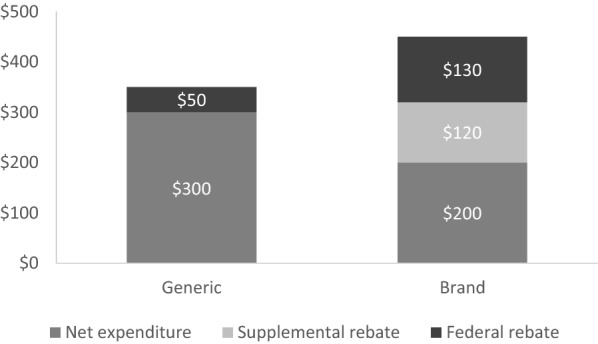
Medicaid expenditures and rebates for a 1-month supply of a generic medication and its brand equivalent. The total of each column represents the Medicaid reimbursement to pharmacies for dispensed medication

Figure 2 illustrates an example of a generic medication and its corresponding brand. In this example, the sum of bars in each column represents Medicaid’s total upfront reimbursement to pharmacies for a 1 month supply of medication. While the reimbursement to pharmacies for the generic medication is lower ($350) than the reimbursement for the brand ($450), the net expenditure by the state, after rebates, is actually higher for the generic medication ($300) than for the brand ($200). For 12 months of therapy for 5000 individuals, for example, the lower net cost of the brand medication results in a savings of approximately $6.0 million. In this example, as with buprenorphine/naloxone in the case above, Medicaid programs will generally prefer brands over generics when brands have a substantially lower net cost to the state.

## Mandates to remove prior authorization

Medicaid programs use prior authorization to establish their preferred drug lists. However, especially in the case of opioid use disorder treatment, providers and patients often experience prior authorization as a barrier to effective care [9–12]. In response, provider and patient advocacy groups have called for removal of prior authorization, focusing on several classes such as opioid use disorder medications as well as antipsychotics and antiretrovirals. Advocates argue that removing prior authorization will increase treatment uptake and the number of providers willing to treat these stigmatized conditions. Although increasing utilization of evidence-based medication treatments will increase program expenditures, many argue that ensuring members have access to, and use, effective treatments is justifiable and consistent with the requirement that Medicaid pays for medically necessary care.

Beyond increases in expenditures due to increases in utilization, mandates to remove prior authorization may also have the unintended consequence of increasing expenditures due to increases in the net unit cost of medications. Without the ability to designate any medications in a therapeutic class as non-preferred requiring prior authorization, states lose bargaining power and manufacturers have little incentive to provide supplemental rebates. For example, in Fig. 2, a mandate to remove prior authorization would result in loss of the supplemental rebate, leading to an increase in the net cost of the brand medication to $320. For the same twelve months of therapy for 5000 individuals, for example, the state’s pharmacy expenditures for the brand medication would increase by approximately $7.2 million. Even when accounting for 90% generic substitution over time, the state’s pharmacy expenditures would still increase by approximately $6.1 million. Most importantly, this increase in pharmacy expenditures occurs without treating a single additional patient.

## Toward judicious and rational use of prior authorization

It is true that the need to obtain prior authorization can create a barrier to timely treatment for opioid use disorder. It is also true that preferred drug lists, created using prior authorization requirements, are currently one of states’ main tools for ensuring appropriate treatment and negotiating favorable supplemental rebates. While mandates to remove all prior authorization are appealing to many, they come at significant cost in terms of lost rebate negotiating power. Similarly, a state approach to managing its preferred drug list solely to limit utilization, obtain higher rebates, and control costs could jeopardize access to effective treatments.

In the case of buprenorphine/naloxone, there are no specific data to suggest that one formulation is superior to another. Further, as prescribers must obtain specific education to obtain the required waiver, the value of a clinical prior authorization to ensure appropriate patient selection is questionable. Therefore, one approach to provide access while preserving states’ negotiating power is to remove clinical prior authorization on the therapeutic class and prefer at least one, but not necessarily all, formulations so that buprenorphine/naloxone can be dispensed without prior authorization. In the case of patient preference or response to treatment, non-preferred formulations of buprenorphine/naloxone would still be available with prior authorization. This arrangement allows for timely access to buprenorphine/naloxone while maintaining states’ leverage in rebate negotiations.

More broadly, in the absence of a fundamental shift in Medicaid payment policy for prescription medications, there are policy options to reduce the burden of prior authorization requirements. These include streamlining and standardizing prior authorization requests and authorizing pharmacies to dispense, and bill for, a limited supply of medication while a prior authorization request is pending. Efforts to streamline prior authorization requests can include establishment of a standard form for all Medicaid members, allowing electronic submission and management of requests, and implementing algorithms to query claims information to determine if a member meets medical necessity criteria based on data already available. More generally, states with Medicaid managed care programs can reduce prescriber administrative burden by implementing a uniform preferred drug list, with aligned medical necessity criteria for all medications, across the entire program.

## Newer strategies to improve access and contain costs

In light of continued cost increases, states are exploring targeted methods to negotiate better rebates. New York State, for example, has a statutory Medicaid prescription medication spending cap [13]. If expenditures are projected to exceed the cap, the Department of Health identifies specific high-cost medications and provides manufacturers with an additional target supplemental rebate amount. If a manufacturer and the Department of Health do not reach a satisfactory agreement, the Department of Health is authorized to impose utilization restrictions for that medication and potentially for the manufacturer’s other products. While promising, the success of this approach is likely to be dependent on state size and so to achieve significant savings, smaller states may need to modify their approach, pool together, or both.

States are also beginning to use alternative payment models for prescription medications. Currently, states receive rebates based on units dispensed, analogous to fee-for-service reimbursement for medical services. Similar to the shift toward value-based payment for medical services, several states are moving toward value-based payments for prescription medications. Louisiana, for example, implemented an alternative payment model for hepatitis C treatment. Through this model, Louisiana Medicaid made sofosbuvir/velpatisvir available without prior authorization to all Medicaid members in return for an agreement with the manufacturer that the state would pay no more than a fixed cost annually regardless of the number of prescriptions dispensed. Other alternative payment models include outcomes-based models, where states’ expenditures are aligned with measures of efficacy and safety such as medication adherence, reduced medical utilization (e.g., hospital or emergency department), improved disease control (e.g., negative urine drug screens), or other reductions in morbidity and mortality [14, 15]. Oklahoma, for example, made oritavancin—a more costly antibiotic than current alternatives—available without prior authorization on the condition that overall medication and hospital costs to the state do not increase (i.e., the state will receive an additional rebate if those costs increase).

## Conclusion

While much of Medicaid pharmacy policy is shaped by federal regulations and cost considerations, all state Medicaid programs maintain clinical advisory committees and individual clinicians remain a powerful voice in informing policy. Clinicians should consider joining a Medicaid pharmaceutical and therapeutics committee, drug utilization review board, or both, to provide input on the preferred drug list and other measures to ensure that members have access to, and receive, effective medication treatments. In addition to their clinical expertise, clinicians can also serve as advocates to ensure that patients’ needs and perspectives are represented.

In summary, concern over high and rising prescription medication expenditures in Medicaid is longstanding. The MDRP, state supplemental rebate negotiations, and clinical considerations expressed by pharmaceutical and therapeutics committees and drug utilization review boards all shape policy in an intertwined manner. Specific to opioid use disorder treatment, efforts to increase treatment should balance access to medication with preserving states’ ability to negotiate lower drug prices.

## Data Availability

Federal cost reporting data are available publicly at Medicaid.gov.

## Abbreviation

MDRP: Medicaid Drug Rebate Program

## References

[1] Medicaid and CHIP Payment and Access Commission. Matching rates. 2019. https://www.macpac.gov/subtopic/matching-rates/. Accessed 25 Aug 2019.

[2] Centers for Medicare & Medicaid Services. Medicaid drug rebate program. 2019. https://www.medicaid.gov/medicaid/prescription-drugs/medicaid-drug-rebate-program/index.html. Accessed 25 Aug 2019.

[3] Baghdadi R. Health policy brief: medicaid best price. Health Affairs. https://www.healthaffairs.org/do/10.1377/hpb20171008.000173/full/. Accessed 25 Aug 2019.

[4] Medicaid and CHIP Payment and Access Commission (2018). Medicaid payment for outpatient prescription drugs.

[5] Park E. Clearing up confusion about the medicaid rebate program: part I. 2019. https://ccf.georgetown.edu/2019/02/11/clearing-up-confusion-about-the-medicaid-rebate-program-part-i/. Accessed 25 Aug 2019.

[6] Roehrig C (2018). The impact of prescription drug rebates on health plans and consumers.

[7] Magellan Rx Management. Medicaid pharmacy trend report. 2018. https://www1.magellanrx.com/documents/2019/03/medicaid-trend-report_2018.pdf/. Accessed 25 Aug 2019.

[8] Centers for Medicare & Medicaid Services. Expenditure reports from MBES/CBES. 2019. https://www.medicaid.gov/medicaid/finance/state-expenditure-reporting/expenditure-reports/index.html. Accessed 25 Aug 2019.

[9] Hartung DM, Johnston K, Geddes J, Leichtling G, Priest KC, Korthuis PT (2019). Buprenorphine coverage in the medicare part D program for 2007 to 2018. JAMA.

[10] Andrews CM, Abraham AJ, Grogan CM, Westlake MA, Pollack HA, Friedmann PD (2019). Impact of medicaid restrictions on availability of buprenorphine in addiction treatment programs. Am J Public Health.

[11] Andraka-Christou B, Capone MJ (2018). A qualitative study comparing physician-reported barriers to treating addiction using buprenorphine and extended-release naltrexone in U.S. office-based practices. Int J Drug Policy..

[12] Kermack A, Flannery M, Tofighi B, McNeely J, Lee JD (2017). Buprenorphine prescribing practice trends and attitudes among New York providers. J Subst Abuse Treat.

[13] The Pew Charitable Trusts. Issue brief: New York's Medicaid Drug Cap. 2019. https://www.pewtrusts.org/en/research-and-analysis/issue-briefs/2018/04/new-yorks-medicaid-drug-cap. Accessed 25 Aug 2019.

[14] Stuard S, Beyer J, Bonetto M, Driver R, Pinson N (2016). Summary report: State Medicaid Alternative Reimbursement and Purchasing Test for High-cost Drugs (SMART-D).

[15] Dworkowitz A, Fiori A, Bachrach D. Extending VBP models into medicaid drug purchasing: challenges and opportunities. 2019. https://www.healthaffairs.org/do/10.1377/hblog20190520.247063/full/. Accessed 25 Aug 2019.

